# A Case of Isolated Elevated Copper Levels during Pregnancy

**DOI:** 10.1155/2011/385767

**Published:** 2011-07-10

**Authors:** LaToya R. Walker, Meghan Rattigan, Joseph Canterino

**Affiliations:** Department of Obstetrics and Gynecology, Jersey Shore University Medical Center, 1945 Route 33, Neptune, NJ 07753, USA

## Abstract

*Introduction*. Outside of Wilson's Disease, abnormal copper metabolism is a rare condition. In pregnancy, excess copper levels can be associated with intrauterine growth restriction, preeclampsia and neurological disease. *Case Report*. A 32 year old Gravida 4 para 2012 with an obstetrical history complicated by elevated copper levels presented for routine prenatal care. Her children had elevated copper levels at birth, with her firstborn child being diagnosed with autism and suffering three myocardial infarctions and being treated for elevated copper levels. During her prior pregnancies, she declined treatment for her elevated copper levels. During this pregnancy, she had declined chelation therapy and instead choose zinc therapy. She delivered a healthy infant with normal copper levels. *Conclusion*. Alterations in copper metabolism are rare, the consequences in pregnancy can be devastating. While isolated elevations of copper in pregnancy is exceedingly rare, it is treated the same as Wilson's disease. The goal is to prevent fetal growth restricting and neurological sequelae in the newborn and preeclampsia in the mother. Counseling, along with treatment options and timely delivery can greatly improve neonatal and maternal outcome.

## 1. Background

Isolated abnormal copper metabolism in pregnancy is a rare condition. If left untreated, it may be associated with developmental sequelae in the newborn. Although copper is a trace mineral and accounts for only 0.01% of total body weight, it plays an important role in electron transport, neurotransmitter synthesis, collagen cross-linkage, and melatonin production and is an important factor in the coagulation cascade. Copper is absorbed by the proximal small intestines and transported to the liver. Toxicity associated with abnormal metabolism, as seen in Wilson's disease, can result in excessive copper accumulation and deposition in many tissues. This can lead to cardiac dysfunction, liver cirrhosis, pancreatic dysfunction and neurological abnormalities [1]. Pregnancy induces little change in the metabolism of this trace metal, only retention in the amounts needed for fetal growth [2]. Abnormal copper metabolism may be associated with intrauterine fetal growth restriction, preeclampsia, and neurological sequelae. We present a case of unexplained abnormally elevated copper levels outside of Wilson's disease only during pregnancy. 

## 2. Case

The patient is a 32-year-old gravida 4 para 2012 who presented for routine obstetrical care at 12 weeks gestation. Prior pregnancies were complicated by two prior cesarean deliveries and elevated copper levels. Postpartum, her copper levels normalized. Evaluation for Wilson's disease, in both the patient and her children, was negative. Her two sons, from different fathers, had elevated copper levels at birth. Her first child was diagnosed with autism and had three myocardial infarctions. He is still being treated for elevated copper levels. Her second child was diagnosed with autism and his copper levels are now normal. During those pregnancies, the patient declined treatment for elevations in her copper.

During the current pregnancy, copper levels were again elevated. She was followed in the high-risk obstetrical clinic. In the first trimester, her copper level was 154 *μ*g/dL, the high end of normal, with normal copper levels ranging from 70–155 *μ*g/dL. As her pregnancy progressed, the levels rose as high as 260 *μ*g/dL with zinc levels decreasing to 61 *μ*g/dL (normal levels of zinc range from 70–150 *μ*g/dL). The remainder of her blood work, including liver function tests, urine drug screens, and complete blood counts were within normal limits. She repeatedly refused treatment with chelation therapy, but instead she opted to increase her intake of zinc. Following zinc supplementation of 100 mg daily, her serum zinc levels normalized to 140 *μ*g/dL. This led to a decreased, but still elevated, copper level of 245 *μ*g/dL.

At 36 weeks, an amniocentesis was performed, which showed lung maturity. A repeat cesarean section was performed. She delivered a liveborn male weighing 2700 grams with Apgar scores were 9 and 9 at 1 and 5 minutes, respectively. Pathological examination of the placenta, membranes and cord were negative for copper. Additional staining for copper was also negative. The infant's blood copper levels and hemoglobin were within normal limits. He showed no evidence of infection; all of his labs and physical examinations were normal. While thyroid function tests were normal, the baby did not have evidence of heart disease during cardiac and NICU evaluation and workup. The newborn has achieved appropriate developmental milestones to date. Maternal serum copper levels returned to normal during the postpartum period. 

## 3. Discussion

Majority of copper is absorbed in the enterocytes of the duodenum and proximal small intestine and incorporated in the liver into apoceruloplasmin, forming ceruloplasmin. Ceruloplasmin represents 90% of circulating copper, and excesses are excreted into bile. Copper participates in multiple enzymatic reactions with varied physiological roles from melanin production to wound healing to electron transport. It stimulates the absorption of iron and is required for the synthesis and function of hemoglobin. It is also involved in the production of elastin and collagen which contribute to the structural stability of bone, cartilage, and tendons [1].

Wilson's disease is an autosomal recessive disorder of copper metabolism with a worldwide prevalence of 1 : 30,000 [3]. The cause of copper elevations is due to an inherited genetic defect in the copper transport which reduces biliary excretion. The protein responsible for copper transport from the liver to bile is ATP7B. Mutation in the allele for this gene leads to absence or diminished function, resulting in decrease copper excretion. As the copper accumulates in the liver, it causes liver disease and damage. Once the capacity of the liver is exceeded, copper diffuses into the bloodstream and deposits into other organs, causing damage specifically to the brain, eyes, and kidneys [1]. Recent studies have shown that during pregnancy, ATP7B plays a role in transporting copper from the placenta to maternal circulation, thus preventing fetal overload. If dysfunctional, excess copper remains in the fetus and placenta leading to oxidative damage resulting in fetal loss or damage [4]. 

While our patient did not have Wilson's disease, abnormal copper metabolism is a rare condition. Workup for other causes of abnormal elevated copper metabolism were negative in this patient; nevertheless, her treatment and management were the same. Excess copper levels can be associated with preeclampsia secondary to excess buildup in the liver, and the fetus can become growth restricted and have neurological sequelae because of oxidative damage caused by copper accumulation in the placenta and fetal tissue [4, 5]. 

There are few case reports in the literature about successful pregnancies in women with Wilson's disease, probably due to hormonal changes secondary to chronic liver disease, endocrine disorders along with infertility, and recurrent miscarriages due to excess copper accumulation in the uterus [3, 4]. However, patients who receive proper treatment can conceive and have a favorable outcome in pregnancy.

 Penicillamine, trientine, tetrathiomolybdate, and zinc are drugs used for the treatment of Wilson's disease. Penicillamine reduces copper levels by chelation and forms a soluble complex that is excreted in the urine [1]. It has been observed in more than 100 pregnancies for a variety of medical conditions including rheumatoid arthritis. While the majority of those pregnancies resulted in healthy newborns, some defects were observed such as vein fragility, impaired wound healing, cutis laxa, low-set ears, micrognathia, and hyperflexion of hips and joints. Counseling and close monitoring is essential to prevent defects in newborn and maintain effective therapy for mother [3, 6]. The alternative, zinc, is usually reserved for maintenance therapy; nevertheless, it appears to be equally effective as penicillamine and easily tolerated [1]. The effects of these treatments suggest that it is relatively safe during pregnancy. However, there are too few exposed infants to be certain.

To our knowledge, case reports of abnormal elevated copper metabolism in pregnancy outside of Wilson's disease are nonexistent. Nonetheless, we treated this patient the same, because the consequences of untreated Wilson's disease in pregnancy can lead to preeclampsia, fetal loss or fetal growth restriction, and neurological damage. In the limited case reports on successful pregnancies complicated by Wilson's disease, penicillamine was the drug of choice and is the mainstay of therapy, and very few case reports used zinc as the only therapeutic option during pregnancy. In our patient, zinc therapy seems to have limited the effects of excess copper to the fetus. And while it is still too early to know whether or not this child will develop autism or any other neurological sequelae, zinc regimen seems to have achieved its purpose, which is a safe maternal and neonatal outcome. Additionally, counseling, along with treatment options and timely delivery, can greatly improve neonatal and maternal outcome. 
